# Development and Validation of a Game for Older Adults on Lifestyles and Frailty

**DOI:** 10.3390/nursrep14030184

**Published:** 2024-09-20

**Authors:** Ana da Conceição Alves Faria, Maria Manuela Martins, José Alberto Laredo-Aguilera, João Miguel Almeida Ventura-Silva, Olga Maria Pimenta Lopes Ribeiro

**Affiliations:** 1 Abel Salazar Biomedical Sciences Institute, University of Porto, 4050-313 Porto, Portugal; mmmartins@icbas.up.pt; 2 Médio Ave Local Health Unit, 4760-412 Vila Nova de Famalicão, Portugal; 3 CINTESIS@RISE, 4200-450 Porto, Portugal; joao.ventura@essnortecvp.pt (J.M.A.V.-S.); olgaribeiro@esenf.pt (O.M.P.L.R.); 4 Facultad de Fisioterapia y Enfermería, Campus de Fábrica de Armas, Universidad de Castilla-La Mancha, Av de Carlos III, nº 21, 45071 Toledo, Spain; josealberto.laredo@uclm.es; 5 Multidisciplinary Research Group in Care (IMCU), University of Castilla-La Mancha, 45005 Toledo, Spain; 6 Northern Health School of the Portuguese Red Cross, 3720-126 Oliveira de Azeméis, Portugal; 7 Nursing School of Porto (ESEP), 4200-072 Porto, Portugal

**Keywords:** healthy aging, lifestyle, frailty, gamification, rehabilitation, nursing

## Abstract

Background: Games are a promising strategy for rehabilitating older adults. The effect of games on promoting healthy lifestyles and preventing frailty remains uncertain. This article aims to describe the process of development and validation of the game “Bem-me-quer para a saúde”^®^ to promote the acquisition of healthy lifestyles and prevent frailty in over 65-year-olds. Methods: This study comprised three distinct phases, spanning from December 2023 to June 2024. The first phase comprised a thorough review of the scientific literature on the frailty and lifestyles of older adults, a second phase of game design, and a third phase of content and semantic validation conducted by specialized nurses and older adults. Results: After revising literature, this study utilized an e-Delphi with a panel of 14 specialist nurses who underwent two rounds of evaluation. The “Bem-me-quer para a saúde”^®^ game includes a puzzle board with 54 pieces and 30 educational cards. In the final phase, we evaluated the game on a cohort of 50 older adults, highlighting its reflective, interactive, and educational aspects. Conclusions: The game presented sufficient evidence of content validity and relevance to clinical practice. We should conduct additional research to evaluate its impact on lifestyle modification and frailty prevention.

## 1. Introduction

Despite the noteworthy increase in the longevity of the Portuguese populace, the quality-adjusted life years gained from life extension still possess the potential for enhancement. In this regard, reevaluating the models for implementing active aging has become a significant challenge for an evolving society [1]. From the viewpoint of successful aging models, the preservation of cognitive and physical functions is a fundamental pillar, along with social participation and general life satisfaction [2,3,4].

Frailty is a multifactorial clinical syndrome that is characterized by a decline in energy reserves and an increase in vulnerability among older adults. This reduces their capacity for homeostatic adaptation to the accumulation of physical, psychological, and social deficits [5]. This condition causes older adults to develop diseases and limitations that affect their independence and autonomy in their daily activities [6,7,8,9].

Recent studies indicate that the risk of mortality for frail older adults is significantly higher than for non-frail older adults [6,8,10,11,12]. Furthermore, through appropriate interventions, we can mitigate or even reverse this condition [13,14]. Despite this, interventions remain underutilized in primary health care [13,14] due to the lack of a standard approach to frailty intervention [14,15]. Furthermore, older adults tend to perceive frailty negatively [16,17], believing it to be inevitable or unchangeable [18].

Several studies indicate that healthy lifestyles are associated with reduced frailty in older adults [19,20]. However, older adults are still not very active [21]. They are reluctant to engage in physical activity; they often find it monotonous, repetitive, unattractive, or unrewarding [22], and they participate little in group and social activities. They also engage in a few health-promoting behaviors [23,24].

It is imperative to discover strategies that involve and inspire older adults to incorporate healthy lifestyles into their daily routines to mitigate the fragility that may arise with aging [25,26].

Adherence and acceptance are crucial to understanding the facilitating factors, difficulties, and concerns of older adults [25,26,27,28]. The use of games has become an important playful intervention strategy. It can be fun, but also pedagogical, involving, and motivating [27,29]. Recent studies have also revealed that games facilitate enhanced control over attention, enhance learning, and equip individuals with a thorough comprehension of the long-term consequences of their actions, thereby facilitating the adoption of behavior modification strategies [30,31,32].

Although digital games are popular, non-digital formats—namely board and card games—have a long history of use in therapeutic settings, with the advantage of facilitating face-to-face interactions with health care professionals and providing important learning opportunities [26,33,34]. Numerous studies link board and card games to physical benefits such as balance or strength training, fine motor skills, memory training, and social participation. These stimuli represent positive and motivating modifications in health-seeking behavior [35,36,37,38,39,40,41]. Consequently, we hold the belief that our contribution to the field has the potential to be significant. Serious games developed with expert input to validate the design, goals, rules, and content [42] have the potential to alleviate the dearth of educational resources addressing healthy lifestyles and early intervention for frailty among older adults. The commitment of older adults to adopting healthy lifestyles depends on their knowledge and the demystification of barriers. Therefore, games are a lively and playful way to capture attention [25,26]. Therefore, this study aimed to describe the process of development and validation of the game “Bem-me-quer para a saúde”^®^ to promote the acquisition of healthy lifestyles and prevent frailty in over 65-year-olds.

## 2. Materials and Methods

In this study, we present a pilot study that outlines the development and validation of a game. This study took place in northern Portugal and consisted of three distinct phases. The initial phase comprised a thorough review of the scientific literature regarding the frailty and lifestyles of older adults; the second phase involved the conception of the game; and the third phase involved the content and semantic validation by specialist nurses and older adults, as recommended by the Pasquali model [43].

### 2.1. Study Design

In the first phase, we reviewed the literature on the subject and adapted and validated the Individual Lifestyle Profile instrument for the Portuguese–European context. We then analyzed the association between lifestyle and multidimensional frailty in community-dwelling older adults using exploratory studies [23,44]. Subsequently, it became imperative to educate older adults on the importance of adopting healthy lifestyles and preventing frailty through educational approaches. Therefore, by examining the dimensions of the “Individual Lifestyle Profile” [44] and “Tilburg Frailty Indicator” [45] instruments, we identified seven thematic fields and incorporated them into the game’s content. In the subsequent phase, the authors developed the game. The game is referred to as “Bem-me-quer para a saúde”^®^ by the authors, which translates to “loves-me, loves-me-not for health”, similar to the game “loves-me, loves-me-not”, commonly associated with the petals of a daisy. The game is a puzzle board game with educational cards, and the game design encompasses the production and registration of the logo, selection of colors, creation of the puzzle board figure, as well as assembly of the cards and the creation of the instruction manual.

The authors selected a game comprising a puzzle board and educational cards, as the objective of the game was to encourage reflection on the present lifestyle and frailty profile and to comprehend the necessity for behavioral modification. During this phase, we examined methods for achieving the therapeutic objectives of the game, its pedagogical approach, and its regulations. To maintain the interest of older adults, the game needed to be vibrant, amusing, and comprehensible.

The game was subsequently validated by a panel of expert specialist nurses. They used an e-Delphi method to assess its content, regulations, and strategies to engage older adults in promoting healthy lifestyles and preventing frailty. The authors employed the e-Delphi technique because of its structured group communication approach. Experts evaluated complex issues characterized by ambiguous and incomplete knowledge through an interactive process. This approach facilitates a quicker, more accessible, and more efficient procedure while simultaneously enhancing the involvement of experts across diverse geographical regions [46,47]. To ensure rigor, this study adhered to the recommendations for the Conducting and Reporting of Delphi Studies (CREDES) [48].

**Figure 1:**
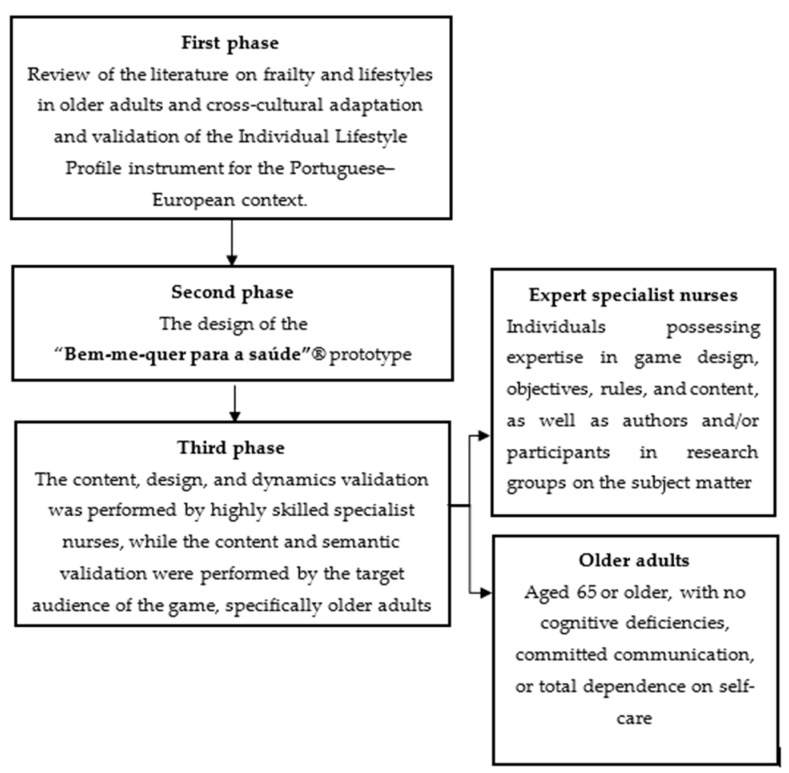
A flowchart with a systematized workflow for the development and validation process of “Bem-Me-Quer para a Saúde”^®^.

After validation by specialist nurses, the game was tested and approved by 50 older adults, as shown in Figure 1.

### 2.2. Participants and Data Collection

The Portuguese population consisted of Portuguese specialist nurses and older adults.

We invited experts to participate in this study via email using a non-probabilistic convenience sampling method, specifically snowball sampling. The criteria for inclusion included being a nurse practitioner with at least 10 years of experience in caring for older adults, having experience in game design including objectives, rules, and content, or having been an author and/or participant in research groups on the topic.

Despite inviting a total of 20 experts, the panel comprised only 14 individuals, as some were unable to meet within the previously established completion deadline. The collection of data for e-Delphi took place between February and April of 2024.

A panel of experts validated the game’s content, design, and dynamics, and then 50 older adults validated it. Inclusion criteria were age 65 or older with no cognitive impairments. We exclude all older adults who are entirely dependent on self-care and exhibit impaired communication skills. As nurses served as mediators in the game, we did not consider a minimum educational requirement for the older adults.

The research team invited and scheduled face-to-face meetings with selected older adults. Between April and June 2024, the researchers explained and utilized the game. A group of volunteers provided the local community facility. At the meetings, the researcher introduced herself to the older adult participants. The researcher further defined the objectives of the meeting, the inherent procedures, and the voluntary nature of their participation.

### 2.3. Instruments

In the first part of the data collection instrument, we briefly characterized the participants by indicating their gender, age, academic degree, and field of specialty. We then requested that they rate the game.

The experts assessed the educational messages conveyed by each theme field. The evaluation included assessing the game’s presentation, graphic design, clarity, relevance, attractiveness, scientific content, organization, formatting, and the relationship between educational cards and puzzle board figures. For evaluation purposes, we utilized a 3-point Likert scale, with 1 representing “disagree”, 2 representing “partially agree”, and 3 representing “completely agree”.

The experts provided an open field to improve the game. We conducted two rounds of evaluation and validation using e-Delphi. All items received a Content Validity Index (CVI) greater than or equal to 0.80, which corresponds to excellence and is a decisive criterion for the item’s importance and/or acceptance [49].

After a panel of experts validated the “Bem-me-quer para a saúde”^®^ game, we invited the older adults to participate and answer a questionnaire that included a sociodemographic characterization and an instrument called the European Portuguese Validation of the System Usability Scale (SUS). John Brooke developed this instrument in 1996; later, Martins et al. validated it for the Portuguese population [50,51]. It has excellent internal consistency (α = 0.80) and allows for evaluating three essential usability components: efficiency, effectiveness, and satisfaction. We chose this 10-question instrument for its ease of use, suitability for small samples with reliable results, and demonstrated construct validity [50,51]. Martins et al. (2015) deem it a comprehensive usability assessment. We divided it into two subscales: usability (items 1, 2, 3, 5, 6, 7, 8, and 9) and learning (items 4 and 10) [50]. To avoid response bias, items 1, 3, 5, 7, and 9 of the SUS are positive, while items 2, 4, 6, 8, and 10 are negative. We utilized a 5-point Likert scale to respond to each inquiry, with responses ranging from strongly disagree to strongly agree. To obtain the general SUS score, the authors utilized the Brooke formula [51]. We subtract 1 from the score of odd questions and 5 from even questions. We then multiplied the total score by 2.5, resulting in a final score of 1 to 100, with a midpoint of 68 [50,51]. Following, we converted the System Usability Scale scores into percentiles (0–100) and graded them (F to A+). Grade A denotes “excellent performance”, Grade C denotes “average performance”, and Grade F denotes “poor performance” [52].

### 2.4. Data Analysis

We conducted statistical analysis and data processing utilizing the Statistical Package for the Social Sciences (SPSS), version 28, employing descriptive and inferential statistics. In the e-Delphi study, quantitative and qualitative analyses were conducted using a three-option Likert scale. For the puzzles and cards, the experts used Bardin’s method [53].

We utilized a 5-point Likert scale and quantitative analysis to assess the game’s usability and satisfaction among 50 elderly individuals. We performed the Kolmogorov–Smirnov (KG) and Shapiro–Wilk (SW) tests to evaluate the normality of the data distribution.

### 2.5. Ethical Considerations

This study followed all recommended ethical procedures, and the Northern Regional Health Administration’s Ethics Committee approved it with Opinion No. 24/2020. All experts provided informed consent through an online form, and all older adults provided informed consent. The game is registered with the Portuguese General Inspection of Cultural Activities under registration number 786/2024, with the National Institute of Industrial Property under national trademark registration number 721547, and with the Portuguese Publishers and Booksellers Association under ISBN 978-989-33-5957-0.

## 3. Results

The research led to the development of a game called “Bem-me-quer para a saúde”^®^. The game’s design and illustrations were created by a professional hired according to the researchers’ guidelines and preferences.

### 3.1. Validation of the Game’s Content with the Experts

After developing the puzzle board and cards, these were sent to 20 specialist nurses. Of those, 14 responded. There was a substantial predominance of specialist nurses, comprising 85.7% female and 14.3% male, with an average age of 34.2 years. In terms of academic qualifications, 14.3% of the respondents possessed a postgraduate specialization degree, 57.1% possessed a master’s degree, and 28.6% possessed a doctorate. The fields of specialty varied from rehabilitation nursing to medical–surgical, community, mental health, and geriatrics. The average length of professional experience was 15.71 years. 

We updated the content on nine educational cards to make the language more accessible to the elderly. We made the phrases easier to read by breaking them down and removing words like “preferably” and “at least”. In the second round of content validation, we simplified the terminology used in the game’s instructions. The game provides important knowledge and has both theoretical and practical value. It can be used by community nurses to educate the elderly about their health ethically and conscientiously. After incorporating the expert’s modifications and recommendations, the vocabulary is now scientific, logical, and suitable for seniors.

Table 1 contains the CVI results.

These measures resulted in the creation of a valid prototype that could be presented to the older adults, who constitute the intended audience for the game.

### 3.2. Game Prototype

**Figure 2:**
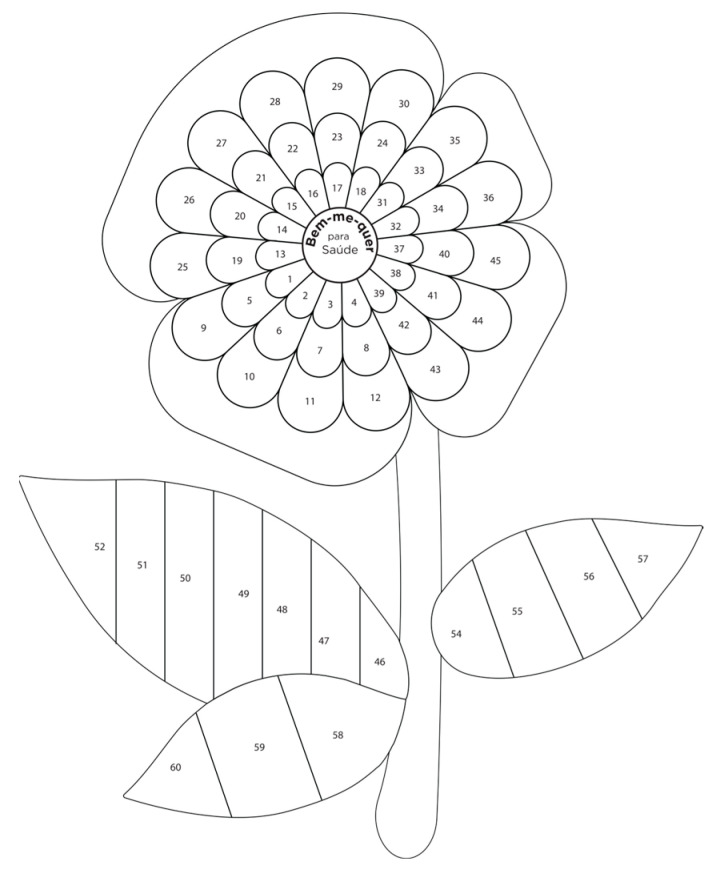
“Bem-me-quer para a saúde”^®^ puzzle board base.

**Figure 3:**
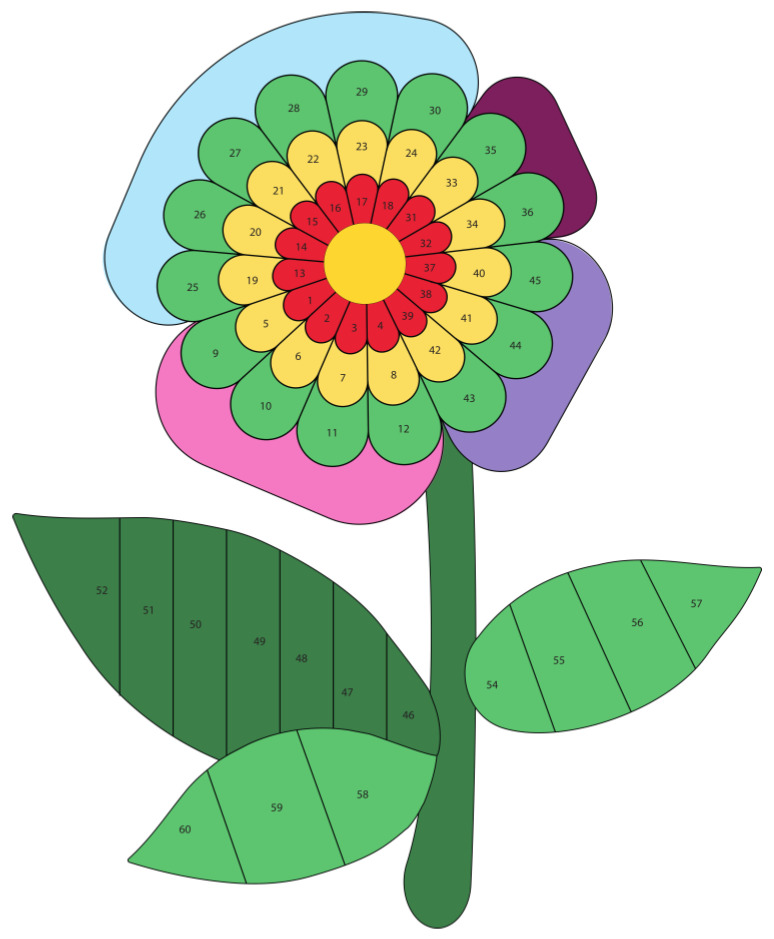
After placing the pieces on the “Bem-me-quer para a saúde”^®^ puzzle board.

**Figure 4:**
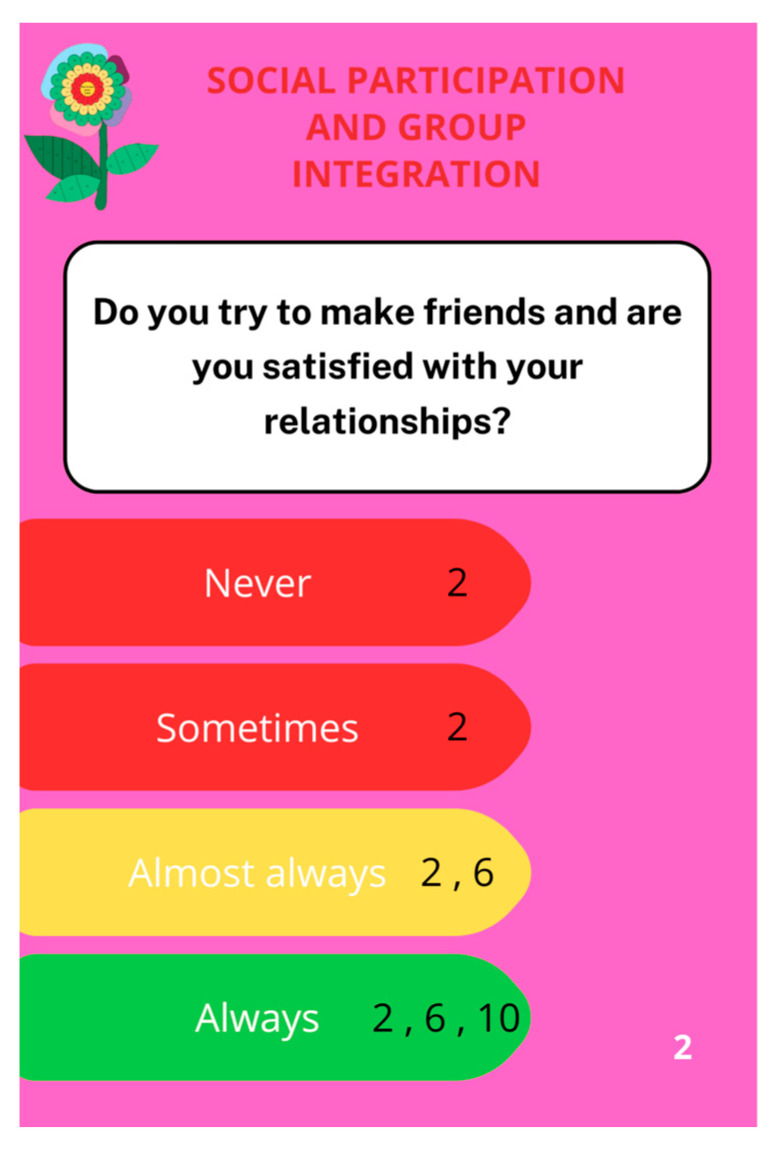
An illustration of a card addressing social participation and group interaction within the “Bem-me-quer para a saúde”^®^ game.

**Figure 5:**
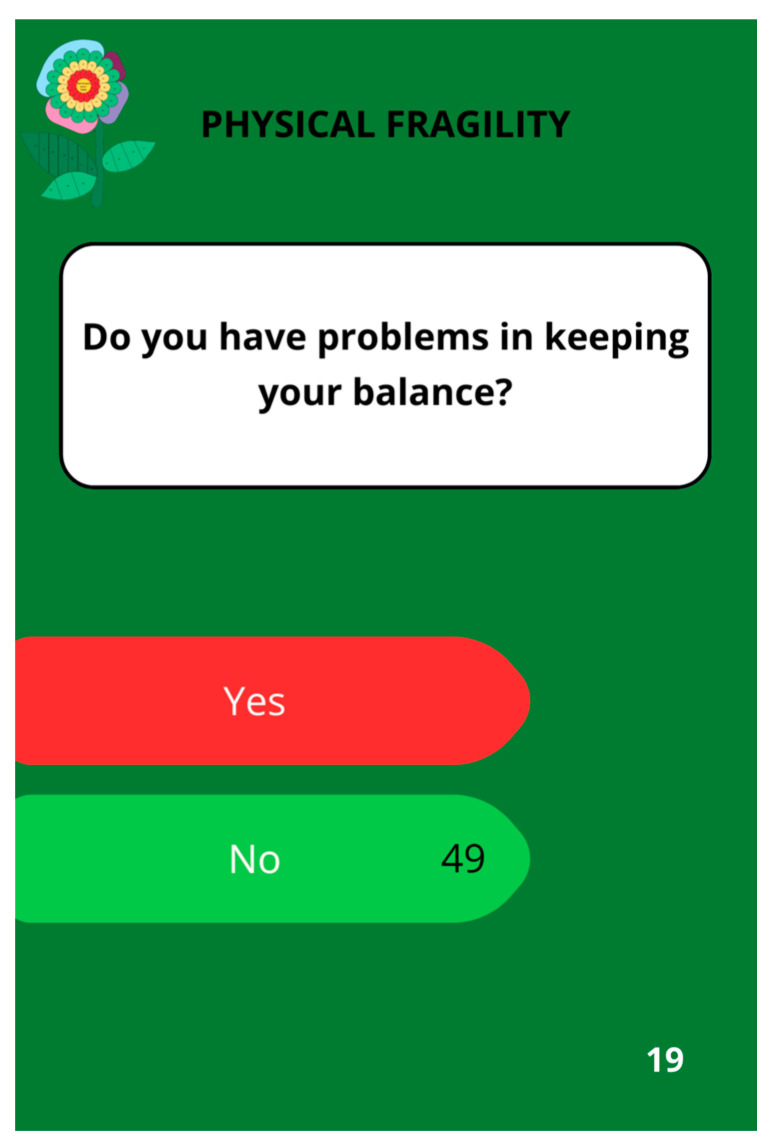
A card dealing with the physical fragility of the “Bem-me-quer para a saúde”^®^ game.

After implementing all modifications, the game design (shown in Figure 2 and Figure 3) and the educational cards’ design (Figure 4 and Figure 5) obtained consensus among the experts.

The game featured a puzzle board with 49 flower petal-shaped pieces, 15 flower leaf-shaped pieces, and 30 educational cards covering 7 domains, namely health self-management (6 blue cards), social participation and group interaction (4 pink cards), citizenship (3 purple cards), and physical activity (2 burgundy cards), physical frailty (8 dark green cards), psychological frailty (4 green cards), and social frailty (3 light green cards). The colors of the larger petals correspond to the colors of the 7 domains discussed in the educational cards.

Each card contained educational messages based on World Health Organization (WHO) and European Commission (EC) guidelines for promoting healthy lifestyles and active aging [54,55].

In the game, experts have found that playing individually with the nurse and the older adult has a positive impact. They explained that the more comprehensive the “Bem-me-quer para a saúde”^®^ approach is, the better the lifestyle and the less frail the individual will be. The game begins when the nurse hands over the center of the flower and its stem. Each older adult then draws a card at random and answers the question on it. Depending on the response, the puzzle is filled in (or not) with the pieces (numbered) indicated on the card. Following, the nurse reads the educational message on the back of the card out loud.

### 3.3. Application and Validation of Game Content with Older Adults

After receiving validation from experts, the game was developed and tested with 50 older adults aged between 65 and 92 years old. Moreover, 88% of the participants were women, 76% were married, 20% were widowed, and 4% were single. An analysis of the dimensions of SUS, particularly usability and learning, was carried out [29]. The values are displayed as minimum, maximum, mean, and standard deviation in Table 2.

We analyzed the theoretical dimensions of the SUS usability and presented the outcomes as mean (M) and standard deviation (SD). According to Table 2, the items Q4 and Q10 presented lower mean scores. On the contrary, Q7 and Q9 exhibited elevated mean scores.

After converting the scores of the 50 SUS using the Brooke formula, we obtained an average score of 90.9 on a scale ranging from 0 to 100. As a result, we rated its ease of use as either superior or excellent. Additionally, we calculated Cronbach’s alpha to measure the internal consistency of the SUS, yielding a value of 0.85.

Based on the analysis of the free-form responses, the following findings emerge as highly valuable and pertinent. Although this result is not specific, it indicates general satisfaction with the game. 

## 4. Discussion

We have created the game “Bem-me-quer para a saúde”^®^ as part of the project “Frail Older Adults at Home: Sensitive Gains to Rehabilitation Nursing Care”. 

The importance of the nurse’s role in the community and the low adherence of the older adults to healthy lifestyles are reasons for developing innovative strategies to promote behavioral change [42]. The WHO and others have reported that this premise is a globally important objective [54,55]. 

As a result, we developed and validated an educational tool that went through various stages with clearly defined objectives to ensure the anticipated outcomes could be achieved safely and effectively [56]. The design of the game “Bem-me-quer para a saúde”^®^ included the esthetics, dynamics, presentation of the rules, linguistic characteristics, and comprehension [42], incorporating a puzzle board game with educational cards.

We chose this format because it is inclusive and easy to use, even for elderly people with limited literacy skills [42]. Furthermore, the game necessitates the acquisition of sensorimotor and cognitive abilities by older adults to complete the puzzle, thereby interfering in other fundamental domains of their life cycle, including physical and adaptive processes [28,57]. 

The game is not meant to replace the intervention of health professionals in the community context; however, it is a motivational strategy for acquiring knowledge, making the process more dynamic and effective. Considering the effectiveness of the teaching–learning process and the management of barriers to adherence to healthy behaviors, we are developing several validation studies of educational technologies in the field of nursing, especially in the community context [58,59,60]. 

We recognized the significance of the initial phase of game design, as it involves analyzing the thematic fields requiring intervention. This includes crafting educational messages that address the specific needs and lifestyles of older adults, following the guidelines provided by the WHO and the EC [54,55]. 

Following the game’s development, experts validated its content, resulting in a CVI of 0.964, considered adequate [49]. This ensures rigor, precision, safety, and validity for the intended objective [61].

After testing the game and obtaining feedback from older adults, we made some changes. However, we realized the changes were unnecessary because the content and dynamics were already suitable for the intended audience. It is important to fix any errors and ensure the game provides the intended experience during this phase [42,56]. 

Furthermore, we used the System Usability Scale (SUS), a commonly used research tool for evaluating how user-friendly a system or product is [50], to analyze the positive feedback of the game. After applying the Brooke formula, the average SUS score obtained was 90.9, considered excellent.

In addition, the results of questions 7 and 9 demonstrated that the older adults were able to quickly learn the game and that it was intuitive. Numerous authors assert that the more users perceive a tool, the greater their commitment to its utilization [62,63,64,65]. Therefore, the game could be used as a complementary health promotion tool with effectiveness, efficiency, and satisfaction in the community context with older people.

Physical games such as board games, puzzles, and cards continue to be an interactive and accessible resource that transcends generational and digital barriers, encouraging older adults to adopt healthy and positive habits in a fun and engaging way [42]. 

Despite the advantages of developing and validating the game, there are still some limitations. These factors comprise the convenience sampling approach and the utilization of a limited group of experts and older adult participants in this study. We have obtained excellent SUS scores, and certain authors have emphasized that a sample of 20 to 30 individuals consistently provides reliable data that can be confidently extrapolated to average SUS scores. Nonetheless, larger samples comprising 50 or more individuals provide enhanced reliability for more precise generalizations. We can reduce the standard error of the mean to represent developmental stages in more complex systems [52,62]. 

Since this was a pilot development and a validation study of a prototype with a small panel of experts and of older adults, and with the goal of validating the game with a larger sample in the future, we decided to test the game with only 50 older adults. If we had a larger sample and applied it to more different regions, the results might have been better [66]. 

Additionally, we have not yet evaluated the medium- and long-term effects of the game. We are interested in conducting a longitudinal study within a health promotion program for frail older adults. We will use the game for the initial evaluation of the program and immediately after its completion. We expect that both the dynamics of the game and the content covered in the program will help us to improve the health-promoting habits of frail older adults.

Despite these limitations, we believe that this study will ensure that older adults are satisfied with the game, based on the comments of older adults who completed the pretest. 

## 5. Conclusions

In this study, we examined the development process for the game “Bem-me-quer para a saúde”^®^. We addressed the game’s content validation and semantic validation. We emphasize the fact that throughout all phases of the methodological framework, experts with expertise in elderly care, authors and/or participants in research groups on the subject, professionals with expertise in game design, and the older adults actively participated in both the development of the game and the evaluation of its utility, thereby enabling adequate validation of this study.

The board game has the potential to empower older adults to adopt healthy lifestyles and behaviors that safeguard against frailty dynamically and interactively. It was possible to strengthen the relationship between the nurse and the older adults, and, in a very positive way, the nurses were able to intervene early to prevent the progression of frailty and negative lifestyles.

## 6. Patents

We registered the game “Bem-me-quer para a saúde”^®^ with the Portuguese General Inspection of Cultural Activities under work registration No. 786/2024, with the National Institute of Industrial Property under national trademark registration No. 721547, and with the Portuguese Publishers and Booksellers Association under ISBN 978-989-33-5957-0.

## Figures and Tables

**Table 1:** The ultimate content validation index as determined by the experts.

Description	CVI
Purposes, goals, or ends to be reached with the game	1
Theoretical significance	1
Practical relevance	1
Design (attractiveness, choice of colors for the board game with a puzzle board and cards)	0.93
Clarity of information (general organization, structure, coherence, presentation, formatting, and size of game pieces)	0.93
Clarity (linguistic characteristics, understanding and style of the game’s writing, and its scientific accuracy)	0.857
Easy for community nurses to use for older adult health education	1
Promoting awareness and behavior change	1
TOTAL GAME CVI	0.964

CVI: content validity index.

**Table 2:** Statistical analysis of the dimensions of the SUS for the “Bem-me-quer para a saúde”^®^ game.

Dimensions	Items	Minimum	Maximum	Mean	Standard Deviation
Usability	Q1. I would like to use this game often	2	5	3.54	0.65
	Q2. I found the game more complex than necessary	1	4	1.81	0.76
	Q3. The game was easy to use	2	5	4.21	0.72
	Q5. I felt that the various functionalities of this game were well integrated	2	5	3.88	0.81
	Q6. I thought this game had a lot of inconsistencies	1	3	1.64	0.58
	Q7. I suppose most people would learn to use this game quickly	4	5	4.50	0.54
	Q8. I found the game very complicated to use	1	3	1.56	0.65
	Q9. I felt very confident when using the game	3	5	4.25	0.62
Learning	Q4. I think I would need the help of a professional to be able to use this game	1	4	1.19	0.92
	Q10. I had to learn a lot before I could handle this game	1	4	1.14	0.93

## Data Availability

The data that support the findings of this study are available from the corresponding author upon reasonable request.

## Public Involvement Statement

There was no public involvement in this research.

## Guidelines and Standards Statement

Recommendations for reporting the results of studies of game development and testing.
